# Targeted Next Generation Sequencing as a Reliable Diagnostic Assay for the Detection of Somatic Mutations in Tumours Using Minimal DNA Amounts from Formalin Fixed Paraffin Embedded Material

**DOI:** 10.1371/journal.pone.0149405

**Published:** 2016-02-26

**Authors:** Wendy W. J. de Leng, Christa G. Gadellaa-van Hooijdonk, Françoise A. S. Barendregt-Smouter, Marco J. Koudijs, Ies Nijman, John W. J. Hinrichs, Edwin Cuppen, Stef van Lieshout, Robert D. Loberg, Maja de Jonge, Emile E. Voest, Roel A. de Weger, Neeltje Steeghs, Marlies H. G. Langenberg, Stefan Sleijfer, Stefan M. Willems, Martijn P. Lolkema

**Affiliations:** 1 Department of Pathology, University Medical Center Utrecht, 3584 CX, Utrecht, The Netherlands; 2 Netherlands Center for Personalized Cancer Treatment, Utrecht, The Netherlands; 3 Department of Medical Oncology, University Medical Center Utrecht, 3584 CX, Utrecht, The Netherlands; 4 Department of Medical Genetics, University Medical Center Utrecht, 3584 CX, Utrecht, The Netherlands; 5 Department of Medical Oncology, Netherlands Cancer Institute, 1066 CX, Amsterdam, The Netherlands; 6 Medical Sciences, Amgen Inc., Thousand Oaks, CA, 91320–1799, United States of America; 7 Department of Medical Oncology, Erasmus MC Cancer Institute and Cancer Genomics Netherlands, 3075 EA Rotterdam, The Netherlands; Baylor University Medical Center, UNITED STATES

## Abstract

**Background:**

Targeted Next Generation Sequencing (NGS) offers a way to implement testing of multiple genetic aberrations in diagnostic pathology practice, which is necessary for personalized cancer treatment. However, no standards regarding input material have been defined. This study therefore aimed to determine the effect of the type of input material (e.g. formalin fixed paraffin embedded (FFPE) versus fresh frozen (FF) tissue) on NGS derived results. Moreover, this study aimed to explore a standardized analysis pipeline to support consistent clinical decision-making.

**Method:**

We used the Ion Torrent PGM sequencing platform in combination with the Ion AmpliSeq Cancer Hotspot Panel v2 to sequence frequently mutated regions in 50 cancer related genes, and validated the NGS detected variants in 250 FFPE samples using standard diagnostic assays. Next, 386 tumour samples were sequenced to explore the effect of input material on variant detection variables. For variant calling, Ion Torrent analysis software was supplemented with additional variant annotation and filtering.

**Results:**

Both FFPE and FF tissue could be sequenced reliably with a sensitivity of 99.1%. Validation showed a 98.5% concordance between NGS and conventional sequencing techniques, where NGS provided both the advantage of low input DNA concentration and the detection of low-frequency variants. The reliability of mutation analysis could be further improved with manual inspection of sequence data.

**Conclusion:**

Targeted NGS can be reliably implemented in cancer diagnostics using both FFPE and FF tissue when using appropriate analysis settings, even with low input DNA.

## Introduction

Sequencing the first human genome in 2008 using massive parallel sequencing was suggested to be the first step in personalized medicine.[1] For clinical decision-making, obtaining genetic information on the entire genome is less suitable due to the high costs, long turnaround time (TAT) and the vast amount of genetic variants with unknown clinical implications. Therefore, simultaneous sequencing of multiple targetable cancer associated genes is gaining popularity. Benchtop Next Generation Sequencing (NGS) platforms and accompanying gene panels are therefore more suitable for routine diagnostics. These platforms provide a cost- and time efficient alternative to classical sequencing techniques like Sanger sequencing.[2, 3] However, sufficiently powered studies providing evidence that NGS is reliable enough to be used in a diagnostic workflow are lacking.

In the routine clinical workup of cancer patients, NGS based techniques need to meet several criteria. The turnaround time between tissue collection and interpretation of sequencing should be short, the NGS platform should be able to handle limited amounts of input material from several sources including formalin fixed paraffin embedded (FFPE) material, sequencing must be deep enough to detect low frequency mutations which may predict therapy resistance.[4]

Currently, two NGS platforms are widely used for diagnostic purposes: the MiSeq/HiSeq/NextSeq (Illumina, Hayward, CA, USA) and the Ion Torrent Personal Genome Machine (PGM) (Life Technologies, Carlsbad, CA, USA).[5, 6] Both platforms could theoretically be implemented for focused gene re-sequencing in routine cancer diagnostics. The MiSeq/HiSeq/NextSeq platform has a higher sequencing capacity and lower costs per base, but requires generally 50–200 ng input DNA which cannot always be obtained from small biopsies[7–9], although alternative library preparation kits are available which use 30 ng DNA.[10] Variant calling of FFPE samples in a clinical setting relies on variant allele frequencies (VAF) of 5–15%,[7, 11, 12] detection of VAF <5% require further validation, and TAT is typically one to two weeks for FFPE samples.[8, 13, 14]

The Ion Torrent platform in combination with Ion AmpliSeq multiplex PCR can use a DNA input as low as 10 ng and the TAT can theoretically be one week.[15, 16] Furthermore, a routine sequencing depth of 500–1,000x can be obtained at costs per sequencing request (e.g. KRAS and NRAS sequencing for colorectal cancer) that are similar to conventional Sanger sequencing[16], and VAF of 2% can be identified reliably.[17–20] Both platforms could theoretically be implemented for focused gene re-sequencing in routine cancer diagnostics, where the choice for one specific platform will depend on the amount of input material, the number of NGS requests and the required TAT.

Currently, standardized NGS kits are available providing every laboratory with the option to perform NGS. Multiple studies have shown that these standardized kits provide reliable sequencing results in routine cancer diagnostics.[12, 17–20] However, a standardized pipeline for annotation and filtering and interpretation of results for clinical decision-making has not been provided. We routinely tested the Ion Torrent PGM benchtop platform combined with a commercial 50 gene Cancer Hotspot Panel on both fresh frozen (FF) as well as FFPE samples to see whether there are major quality differences between these sample types, explored the lower DNA input limits and validated the results obtained to establish its value for diagnostic use in the routine workup of cancer patients.

## Materials and Methods

### Patient Selection

First, 250 FFPE samples were collected from the archives of the University Medical Center Utrecht, of which 135 samples were retrospectively and 115 prospectively collected. The retrospective samples consisted of both mutated and wildtype samples as called by conventional methods in routine pathology diagnostics. These conventional sequencing analyses consisted of the following techniques to identify mutations in several genes and exons: KRAS exon 2 & 3 and EGFR exon 19–21 using the High Resolution Melting technique, BRAF V600 by Cobas analysis, and TP53 exon 4–9, CTNNB1 exon 3, cKIT exon 9 & 11, PDGFRa exon 12 & 18 by means of Sanger Sequencing (S1 Table). The prospective samples consisted of all mutation analysis requests in our laboratory for a period of 3 months, in total 115 samples. The 250 samples included 23 normal tissues (either normal tissue adjacent to the tumours in the same tissue block or from another tissue block from the same patient) and 227 tumour samples including 15 clonality requests to determine whether several tumours in one patient had a common origin, of which 8 showed a clonal relation between the tumours (see S1 Fig and S2 Table for tissue distribution).

**Fig 1:**
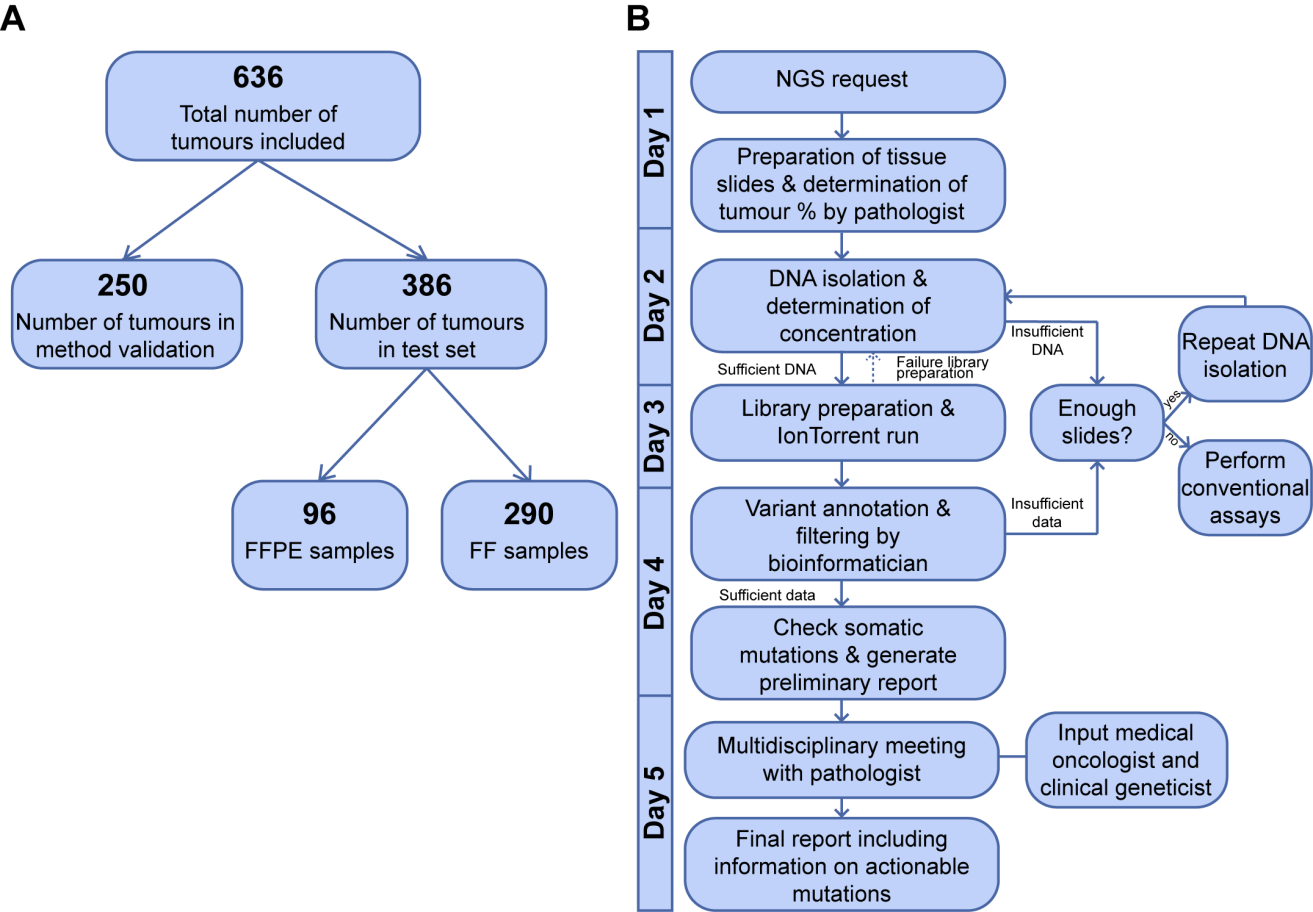
Next Generation Sequencing workflow for routine diagnostics. A) Number of samples included in this study. B) Ion Torrent NGS workflow analysis by which routine diagnostics from tissue arrival to reporting results to the clinician is performed within 5 working days. First, tumour percentage is determined in control sections, and subsequently tumour tissue is macro dissected, DNA is isolated and NGS is performed. If insufficient DNA is isolated and no further tissue is available, conventional techniques like Sanger sequencing is performed. Somatic variants are identified by the Torrent Variant Caller supplemented with variant annotation and filtering. Variants are manually checked in IGV and discussed in a multidisciplinary meeting with necessary clinicians. Finally, results are reported to the responsible clinician.

Next, after completing the validation of the Ion Torrent NGS method of the 250 samples described above, NGS was performed successfully for another 386 samples, of which 290 fresh frozen (FF) and 96 non-paired FFPE tumour samples (see Fig 1A for overview of sample numbers). FF samples were analysed to determine eligibility for enrolment into trials for targeted therapies of the Center for Personalized Cancer Treatment (CPCT; http://www.cpct.nl/en.aspx), while FFPE samples were submitted for routine pathology diagnostics. Written informed consent was obtained from all patients contributing FF tumour samples for one of the CPCT trials and data from FFPE samples was used anonymously. Institutional Review Board approval was obtained and research was carried out in accordance with the ethical guidelines of the Foundation Federation of Dutch Medical Scientific Societies.

NGS was performed according to the flowchart depicted in Fig 1B and is described in more detail below. The complete NGS process from NGS requisition up to reporting of the NGS findings could be completed within 5 working days.

### Sample Preparation

For FFPE samples, tissue was fixed in PBS buffered formalin and embedded in paraffin. A 5 μm section was H&E stained for routine pathology diagnostics. Alternatively tissue was fresh frozen.

Upon arrival of both the NGS requisition and FF or FFPE tissue, 5 μm thick H&E sections were prepared, tumour percentage was determined by an experienced and dedicated pathologist (SMW) trained in determining tumour percentage, and the most tumour rich area was encircled for macro dissection. Minimal input was 1 cm^2^ of a 5 μm tumour tissue section and a minimal tumour percentage of 10% for FFPE. DNA was isolated using the Cobas method (Roche). DNA concentration was determined using Qubit Fluorometer (Life Technologies).

For fresh frozen tissue, biopsies containing more than 30% tumour were selected based on H&E staining by experienced and dedicated pathologists, and DNA was isolated using the NorDiag Arrow (Isogen Life Sciences) as described by the manufacturer.

For FF and FFPE samples, a total of 10 and 20 ng of input DNA was used, respectively, in a final volume of 12 μl. If the DNA concentration appeared to be too low and if tissue was still available, additional DNA was isolated. If there was no remaining tissue, conventional mutation analysis techniques, like sanger sequencing, were performed for FFPE samples.

### Next Generation Sequencing

The Ion Torrent Library was prepared using the Ion AmpliSeq Cancer Panel for the validation study (n = 250) and the Ion AmpliSeq Cancer Hotspot Panel v2 (Life Technologies) for the remaining samples (n = 386). The latter allows for simultaneous amplification of 207 amplicons in hotspot areas of 50 oncogenes and tumour suppressor genes (46 genes present in AmpliSeq Cancer Panel supplemented by 4 genes of interest being *EZH2*, *IDH2*, *GNA11* and *QNAC*). PCR was performed in 17 cycles for FF samples and in 20 cycles for FFPE samples. Samples were barcoded using IonXpress Barcode Adapters (Life Technologies) to allow for discrimination between samples within a NGS run. The DNA concentration of the samples within one sequencing run were normalized using the Qubit 2.0 fluorometer (ThermoFisher Scientific) or the Ion Library Equalizer kit. The Ion AmpliSeq Library Kit 2.0 (Life Technologies) was used for library preparation. The library was mixed with Ion Sphere Particles (ISPs) and the subsequent emulsion PCR and enrichment were performed using the Ion PGM^TM^ Template OT2 200 Template Kit and the Ion One Touch 2 instrument (Life Technologies). Sequencing was performed using the Ion PGM^TM^ Sequencing 200 kit v2 using the Ion 316^TM^ or 318^TM^ chip (Life Technologies) (maximum number of samples on 316 and 318 chip were 6 and 12 respectively). FF and FFPE samples were run on separate chips. Samples were run on the Ion Torrent PGM System^TM^ (Life Technologies) as described by the manufacturer.

### Data Analysis

Sequencing results of the Ion Torrent PGM run were presented via the Torrent Browser, a web-based user interface on the Torrent Server. A Torrent Browser run report contains statistics and quality metrics for the run, such as the Ion Sphere™ Particle (ISP) density, percentage of polyclonal ISPs (ISPs carrying clones from two or more templates), low quality percentage (percentage of ISPs with a low or unrecognizable signal), and percentage of usable reads (the percentage of Library ISPs that pass the polyclonal, low quality, and primer dimer filters). These statistics were used to evaluate the quality of the Ion Torrent PGM run. A good quality run has at least 30% ISP loading and 30% usable reads. A run will not be rejected based on these quality metrics. Instead, individual samples are evaluated as described below.

Reads generated were aligned using the Torrent Mapping Alignment Program (TMAP). This program uses a series of mapping algorithms to map sequence reads to the human reference genome build 19 (hg19). TMAP has been developed to meet Ion Torrent data mapping challenges, such as miscalling homopolymer stretches and increasing read lengths over time. It provides a fast and accurate aligner through the integration of a novel alignment algorithm and three popular algorithms: BWA-short,[21] BWA-long,[22] SSAHA,[23] and Super-maximal Exact Matching.[24] The final alignment of each library is stored in a BAM file.

After the alignment step, coverage statistics were generated using Coverage Analysis plugin version 3.6 (Life Technologies). This plugin takes the TMAP output and a file containing the target regions of CHPv2 as an input to provide statistics per library such as the mean depth of coverage, number of mapped reads, and on-target percentage (percentage of mapped reads which are aligned to the target region). These statistics were used to evaluate the quality of each library in the run. A good quality library has at least a mean depth of coverage of 800x, 80% on-target percentage and 100,000 mapped reads.

The Torrent Variant Caller (TVC) plugin version 3.6 is a genetic variant caller for the Ion Torrent Sequencing platform (Life Technologies) and is used to call somatic single-nucleotide polymorphisms (SNPs), multi-nucleotide polymorphisms (MNPs), insertions, deletions, and block substitutions. The TVC plugin operates on TMAP generated BAM files and requires the following as input: a target region file containing the chromosome regions of CHPv2, a hotspot file containing a list of positions in the human genome and parameter settings file (see S3 Table for TVC parameter settings).

A standardized pipeline was constructed to process each variant detected by the TVC. This pipeline uses a comprehensive Perl Application Program Interface (API) providing efficient access to the Ensembl Variation database.[25] For each variant, this pipeline adds annotations like consequence type (e.g. missense variant), references to other databases (e.g. Unigene, RefSeq, OMIM, Cosmic), biotype of the transcript (e.g. protein coding), amino acid change caused by the variant, gene description. It also provides variant effect scores (SIFT and PolyPhen but these were not used in further evaluation of the variants.

The pipeline also filters out variants that are not included for further evaluation like synonymous, 5’ and 3’ UTR, and intronic variants, coverage <100x and VAF <5%. Finally, probable germline variants (determined using public databases dbSNP, 1000 Genomes and GoNL) and common TMAP or TVC artefacts were filtered out. As an output of the pipeline, a list of variants of each library including all annotations was generated.

### Reporting NGS Findings

Variants annotated and filtered as described above were manually checked by well trained technicians and experienced molecular biologists using IGV (Integrative Genomics Viewer).[26] Variants were checked for reads being >500x, mutant reads exceeding 30x and whether the variant was not in a homopolymer stretch. Furthermore, all requested genes (e.g. RAS and BRAF for colon tumours) were manually checked in IGV as extra check and all amplicons in TP53, cKIT exon 9 and 11, PDGFRa exon 12 and 18 and EGFR exon 19 and 20 were manually checked since large deletions can be missed by the TVC version 3.6. Somatic mutations and variants of unknown significance were noted in a preliminary reporting form. The preliminary report was discussed in a multidisciplinary meeting involving a pathologist and clinicians to enable a determination of the clinical significance of variants that were identified with more background information on the tumour type and medical history of the patient, resulting in a final set of variants that was reported in the final report with a clinical annotation of their proposed significance. Moreover, low frequency variants (VAF <5% and coverage <100x) and variants of unknown significance (usually outside but very near to known hotspot regions) which were identified were discussed. If required, a medical oncologist was consulted to discuss potential treatment options. Furthermore, in case of potential germline variants a clinical geneticist was consulted. The identification of potential germline variants was only based on sequence analysis of the tumour, where population genome sequences were consulted to differentiate between germline SNPs and pathogenic variants. Within 5 working days, the final report including information on potentially actionable mutations was then sent to the responsible clinician.

### Statistical Analysis

Statistical analysis is performed using R. Significant differences are calculated by means of an independent t-test for sequencing run- and library statistics and chi-squared analysis for comparison of base substitutions between FF and FFPE samples. A P value less than 0.05 was considered to be statistically significant.

### Sequence Data

All sequence data described in this manuscript can be found in the European Nucleotide Archive (ENA). Validation set accession number is: PRJEB11579 (http://www.ebi.ac.uk/ena/data/view/PRJEB11579). Test set accession number is: PRJEB11475 (http://www.ebi.ac.uk/ena/data/view/PRJEB11475).

## Results

### Performance of NGS on DNA Samples from Fresh Frozen and Formalin Fixed Material

**Fig 2:**
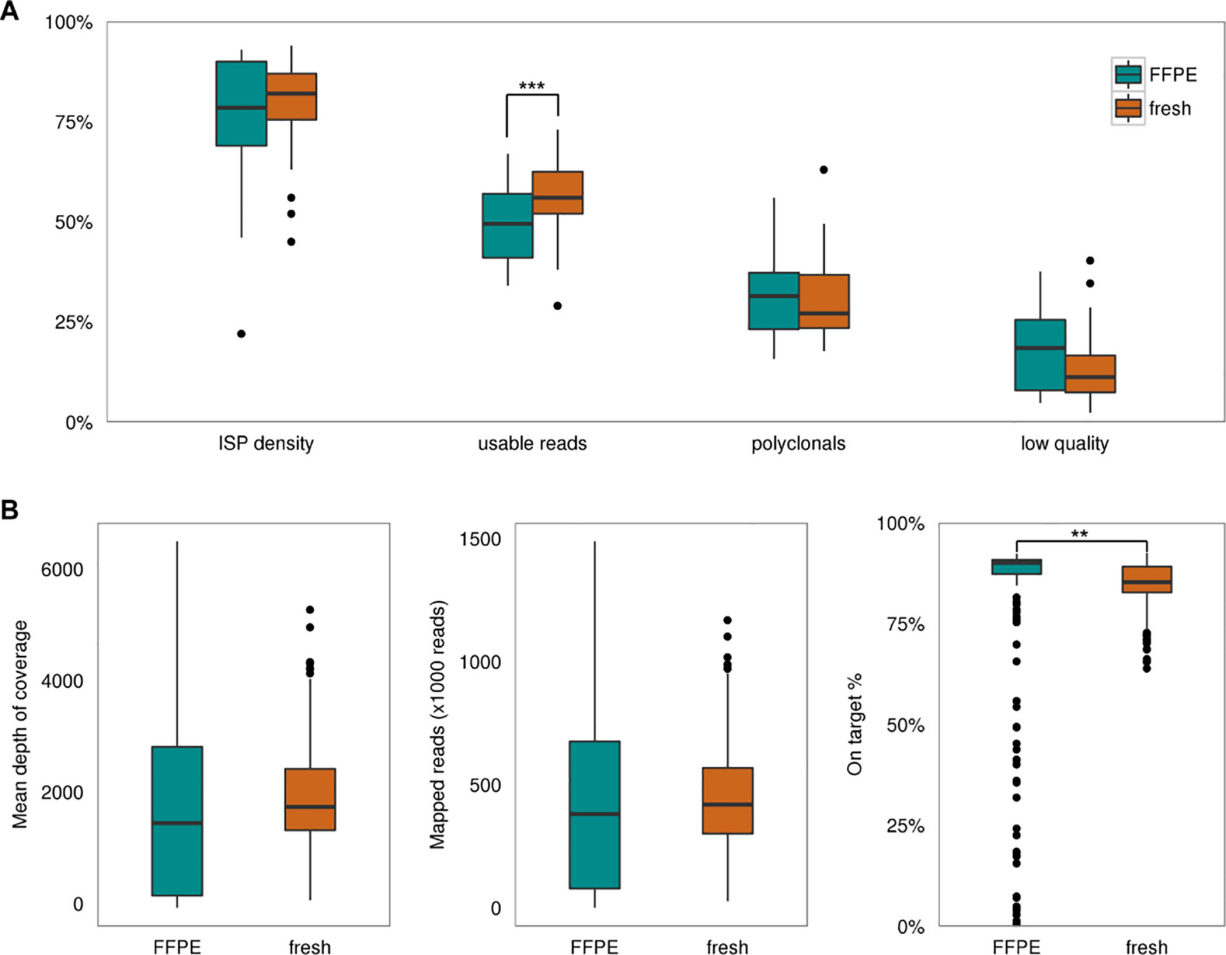
Run and library statistics. A) Boxplot of run statistics of FFPE (green) and FF (orange) samples for 4 variables: 1. the percentage of ISP (Ion Sphere Particle) density (the addressable wells on the chip which have detectable loading); 2. usable reads of the total number of reads (percentage of ISPs that pass the polyclonal, low quality, and primer dimer filters); 3. polyclonals, ISPs that contain more than one template sequence per ISP and 4. low quality, ISPs with a low or unrecognizable signal. The upper and lower “hinges” of the boxplots correspond to the first and third quartiles (the 25^th^ and 75^th^ percentiles). The upper “whisker” extends from the hinge to the highest value that is within 1.5*IQR of the line, where IQR is the inter-quartile range (the distance between the first and third quartiles). The lower “whisker” extends from the hinge to the lowest value within 1.5*IQR of the hinge. Data beyond the end of the vertical lines are outliers and plotted as points. B) Library statistics of FFPE (green) and FF (orange) samples; the mean target base read depth (including non-covered target bases); the number of reads mapped to the full reference genome; and the percentage of mapped reads which are aligned to the target region. Significant differences calculated by means of an independent t-test between FFPE and FF samples are depicted with ** p = 0.002 or ***p = 0.0009).

Sequence runs containing only FF samples resulted in significantly more usable reads (p = 0.0009), defined as reads that passed quality filters (Fig 2A), although the absolute difference in usable reads was only 7.1%. Analysis of library statistics showed a significantly increased percentage on-target reads (p = 0.002) for FF samples compared to FFPE samples (Fig 2B), where the number of samples containing a low percentage on-target reads was limited. Moreover, the samples with low percentage on-target and thus a low coverage could easily be identified: in total 7.7% of the samples were excluded due to a mean coverage <800x of which 71% showed also <80% on-target. These excluded samples consisted for 98% of FFPE samples. The remaining quality parameters including the number of mapped reads did not show differences. Furthermore, all targeted regions could be covered adequately, as none of the amplicons showed an average mean coverage below 100x leading to exclusion from analysis and 87% of the amplicons for FFPE samples and 94% of the amplicons for FF samples were covered >800x on average (S2 Fig). During the 1,5 year intake period of this study, the sequence runs performed at a stable level (S3 Fig), with only a slight decrease of the percentage of usable reads and an increase in the percentage low quality ISPs from the moment of inclusion of FFPE samples half-way this time period.

In summary, a good quality sample could be recognized by a mean coverage of at least 800x and >80% on target.

When comparing coverage of all amplicons in the Ampliseq Cancer Hotspot Panel v2 between FFPE and FF samples, a decreased coverage for the longer amplicons was seen in FFPE samples (S4 Fig). There was also no significant difference in the ratio of C > T or G > A base transitions in the FFPE samples compared to the FF samples (S5 Fig).[27, 28]

### Defining the Requirements for Mutation Calling in DNA Samples

To determine the variant detection limit of the assay, dilution experiments of four FF DNA samples with known *TP53* mutations were performed. With an R squared of 94.53% the dilution data were close to the expected allele frequencies (fitted line, S6 Fig). The known *TP53* mutations were reliably detected down to an allele frequency of 1%. As dilution assays may overestimate the sensitivity of the assay a cut-off of 5% allele frequency was therefore set to be reliable for future diagnostic use.

**Fig 3:**
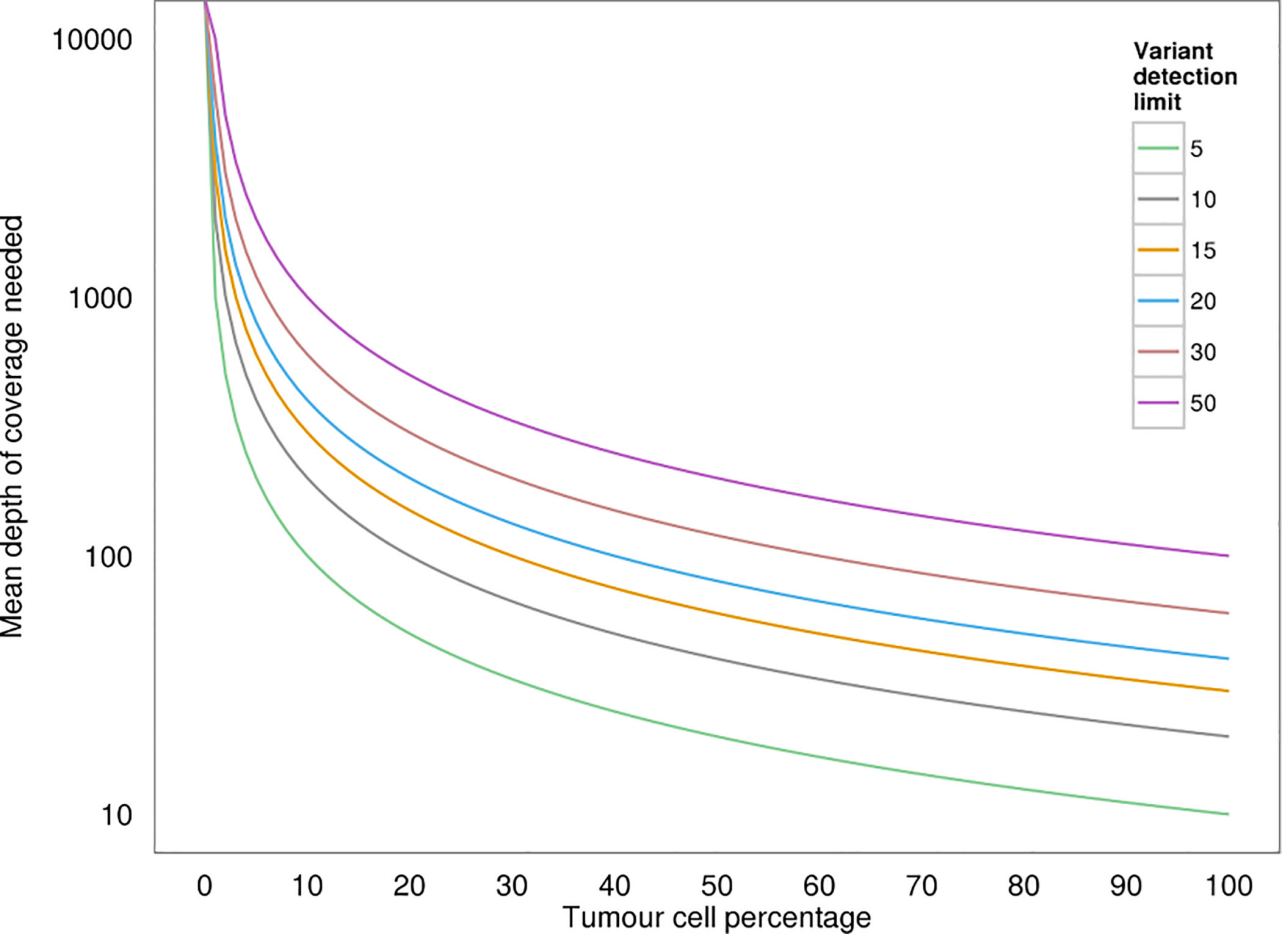
Theoretical model of the detection sensitivity for different variant detection limits. Lines depict the coverage needed for a certain tumour percentage. In this study a detection limit of 20 variants was used, which, combined with a tumour percentage of at least 10%, leads to a needed coverage of 800x.

**Fig 4:**
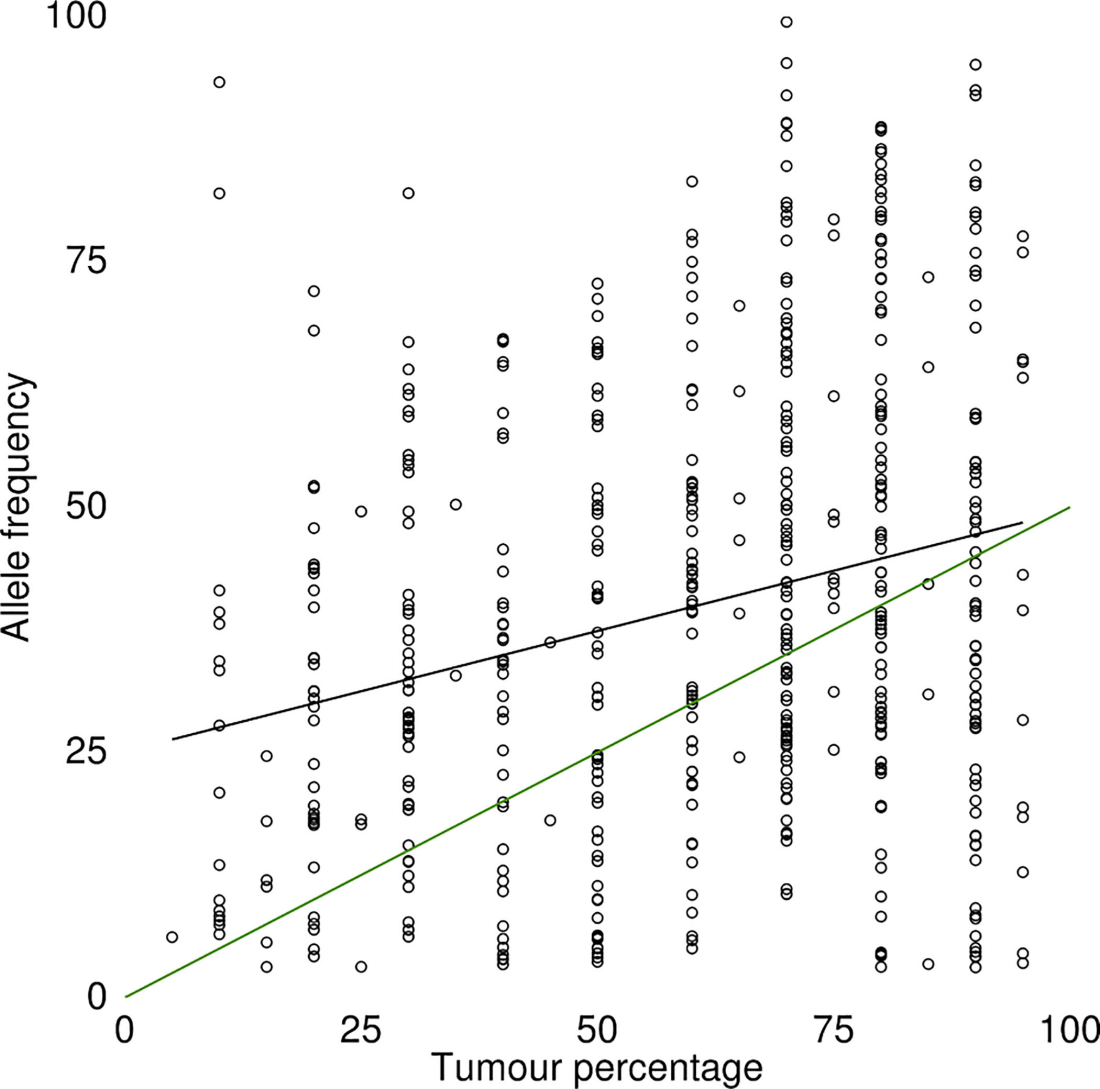
Correlation between tumour percentage and allele frequency. The observed allele frequency for all variants detected using NGS is plotted against the tumour cell percentage as determined by a pathologist. The green line depicts the theoretical line of expected allele frequency of a heterozygous (somatic) mutation versus tumour cell percentage. A forced linear regression line (black line) was plotted to determine whether increased tumour percentage affects the mean allele frequency detected with a correlation coefficient of 0.041.

Since the percentage of tumour cells present in the material used for DNA extraction is an important variable defining the ability of any assay to detect somatic mutations in diagnostic specimens, [29] we predicted that a variant could be detected when at least 20 reads were detected with a coverage of 800x for the amplicon, given the input material contained at least 10% tumour cells (Fig 3). For standard mutation calling, 800x is probably not necessary but our assay was designed to obtain a high sensitivity even for samples with low tumour cell percentages. Next, we performed an analysis on the entire dataset to assess whether tumour cell percentage of the input material affected the mean VAF. Theoretically, a heterozygous mutation in a diploid sample with 10% tumour cells can be reliably detected when using a detection limit of a frequency of 5%, but we did not find a relationship between tumour percentage and VAR (Fig 4).

### Validation of Mutational Profiles Obtained with the Ampliseq Assay

**Fig 5:**
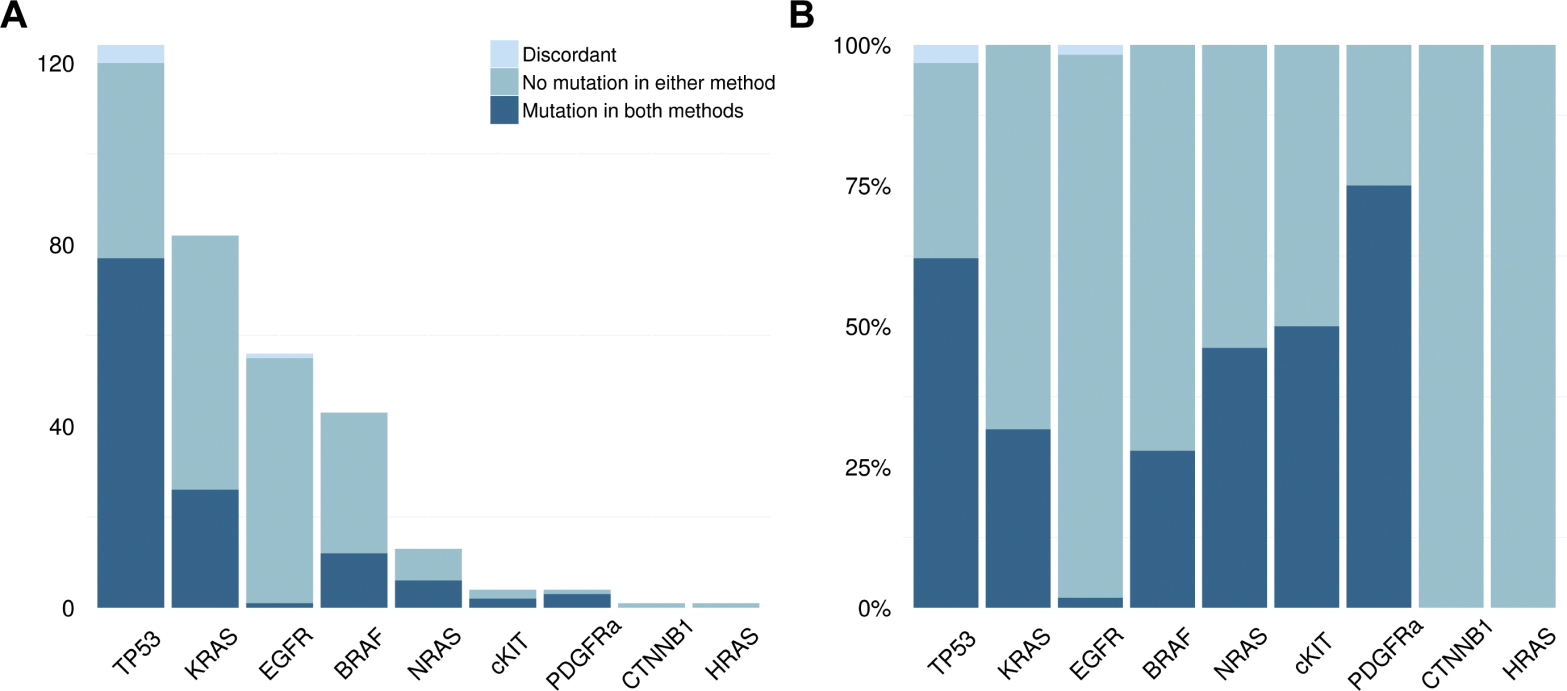
Validation of Next Generation Sequencing. A) The absolute number of samples with a mutation in various genes as denoted on the x-axis that were used for the validation of NGS by means of the Ion Torrent platform. All samples are colour coded: dark blue are the concordant samples with the same mutation in standard versus NGS, the intermediate blue are the concordant samples showing no mutation, the light blue bar represents the discordant samples B) The same data as depicted in Fig 5A, however represented as percentages of all tested samples for a given gene.

We validated 328 variants, of which 323 were concordant between NGS and the conventional techniques, resulting in an overall concordance of 98.5% (sensitivity of 99.1%)(Fig 5, Table 1). Of the 5 discordant samples, two false negative variants of *TP53* exon 8 (p.G266E) were identified using Sanger Sequencing but not using NGS, a discrepancy that could not be resolved. A third false negative variant was identified in *TP53* exon 7 (c.757_758insA, p.T253fs*11) that was not called by TVC but was clearly visible in IGV. The only false positive variant was *TP53*, exon 7 (c.723delC, p.C242fs*5) which was called by TVC but was not visible in IGV upon manual check. The final discordant variant was identified in *EGFR* exon 21 (p.L858R) with an VAF of 7.3% which was not detected using HRM analysis due to the low tumour cell percentage of the input material (estimated at 5–10%). *TP53* is not fully covered in the Ampliseq panel resulting in 19 samples where a *TP53* variant was identified with Sanger Sequencing, which could not be identified using NGS (S4 Table). These data support the conclusion that the Ion Torrent AmpliSeq workflow is a reliable technique for mutation analysis and manual checks in IGV further improve its reliability.

**Table 1:** Concordance between conventional techniques and Ion Torrent based NGS analysis.

	#	Description	Gene	Exon	Variant
	323	Concordant	NA	NA	NA
False negative	2	Discordant	2x TP53	exon 8	G266E
False negative	1	Not called by IT, visible in IGV	1x TP53	exon 7	c.757insA
False positive	1	Called by IT, not visible in IGV	1x TP53	exon 7	c.723delC
	1	IT more sensitive than conventional technique	1x EGFR	exon 20	L858R

For discordant samples information on the variants is provided including a description why the discordance appeared. Only discordant variant *TP53*, G266E, could not be explained; it was visible with Sanger sequencing and could not be identified in the Ion Torrent data. Other discordances could be explained by discrepancies between Ion Torrent software and IGV or by the increased sensitivity of the Ion Torrent compared to the conventional technique in low tumour percentage samples.

### Interpretation of Data Obtained with the Ampliseq Assay

**Fig 6:**
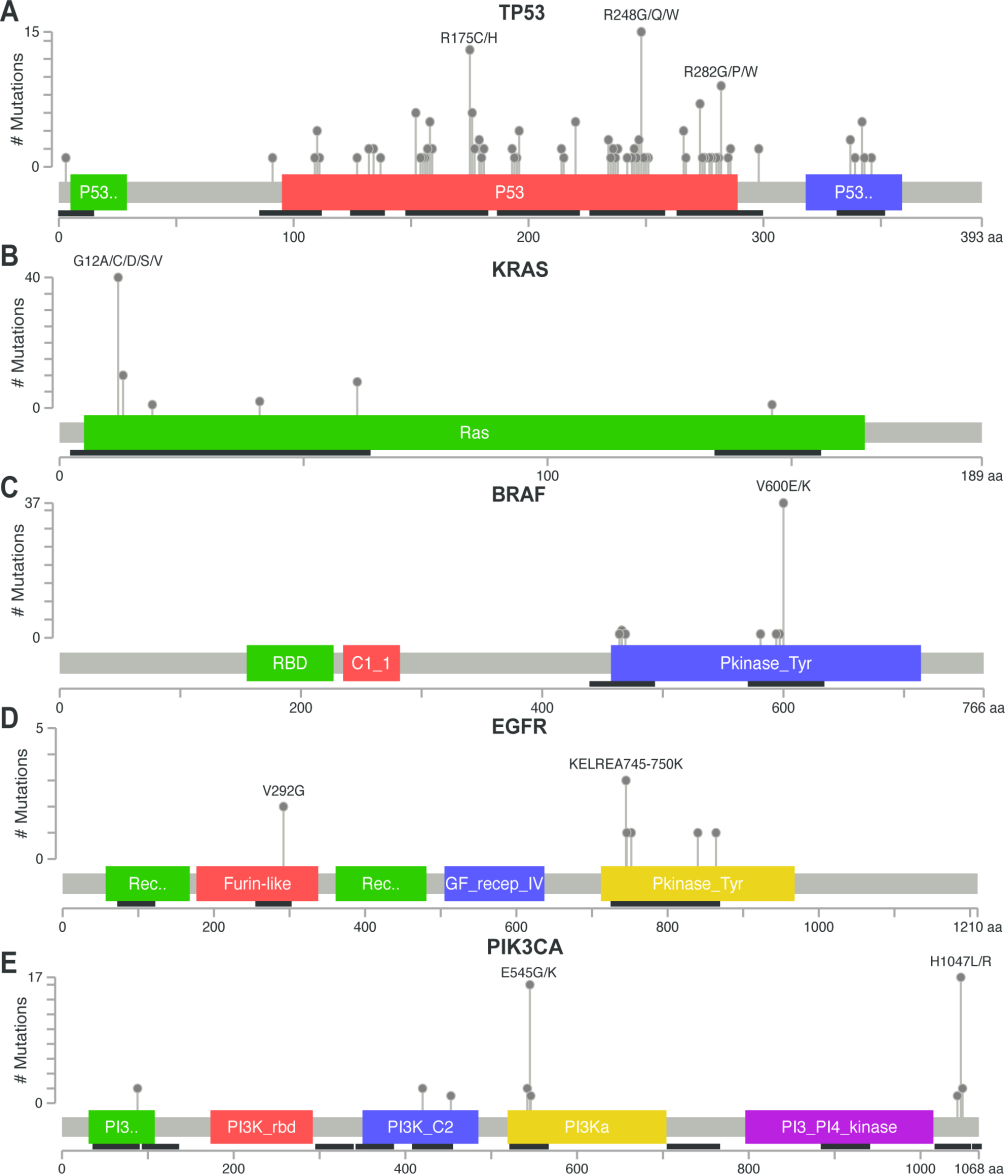
Variant distribution per gene. All graphs depict a lollipop plot (adapted from (Vohra and Biggin, 2013)) showing identified variants relative to a schematic representation of the gene. Any position with a mutation obtains a circle, the length of the line depends on the number of mutations detected at that codon. The grey bar represents the entire protein with the different amino acid positions (aa). The coloured boxes are specific functional domains. On top of the lollipops the most frequent variants are annotated as the amino-acid change at that specific site. Black lines underneath the grey box indicate the regions where the Ampliseq panel covers the gene. A) Mutations identified in the *TP53* gene using NGS, B) *KRAS*, C) *BRAF*, D) *EGFR* and E) *PIK3CA*.

To further understand whether NGS results reflect an expected mutational pattern we analysed all identified mutations in the *TP53*, *KRAS*, *BRAF*, *EGFR* and *PIK3CA* genes in a final dataset containing 386 samples, 290 derived from FF material and 96 derived from FFPE material. Even though the AmpliSeq panel does not cover the entire *TP53* gene, mutations were identified throughout the targeted region (Fig 6A). Comparison with the TCGA database shows a 82% overlap of our findings compared to the TCGA database (S8 Fig). As could be expected, a limited mutation distribution was identified for *KRAS*, *BRAF*, *EGFR* and *PIK3CA* (Fig 6B–6E) as these genes contain mutational hotspot locations, which could be detected reliably in this assay. Of interest, several parts of the *PIK3CA* gene were sequenced without identifying mutations, suggesting an absence of a systematic bias towards false positive findings based on the choice of amplicons sequenced.

**Fig 7:**
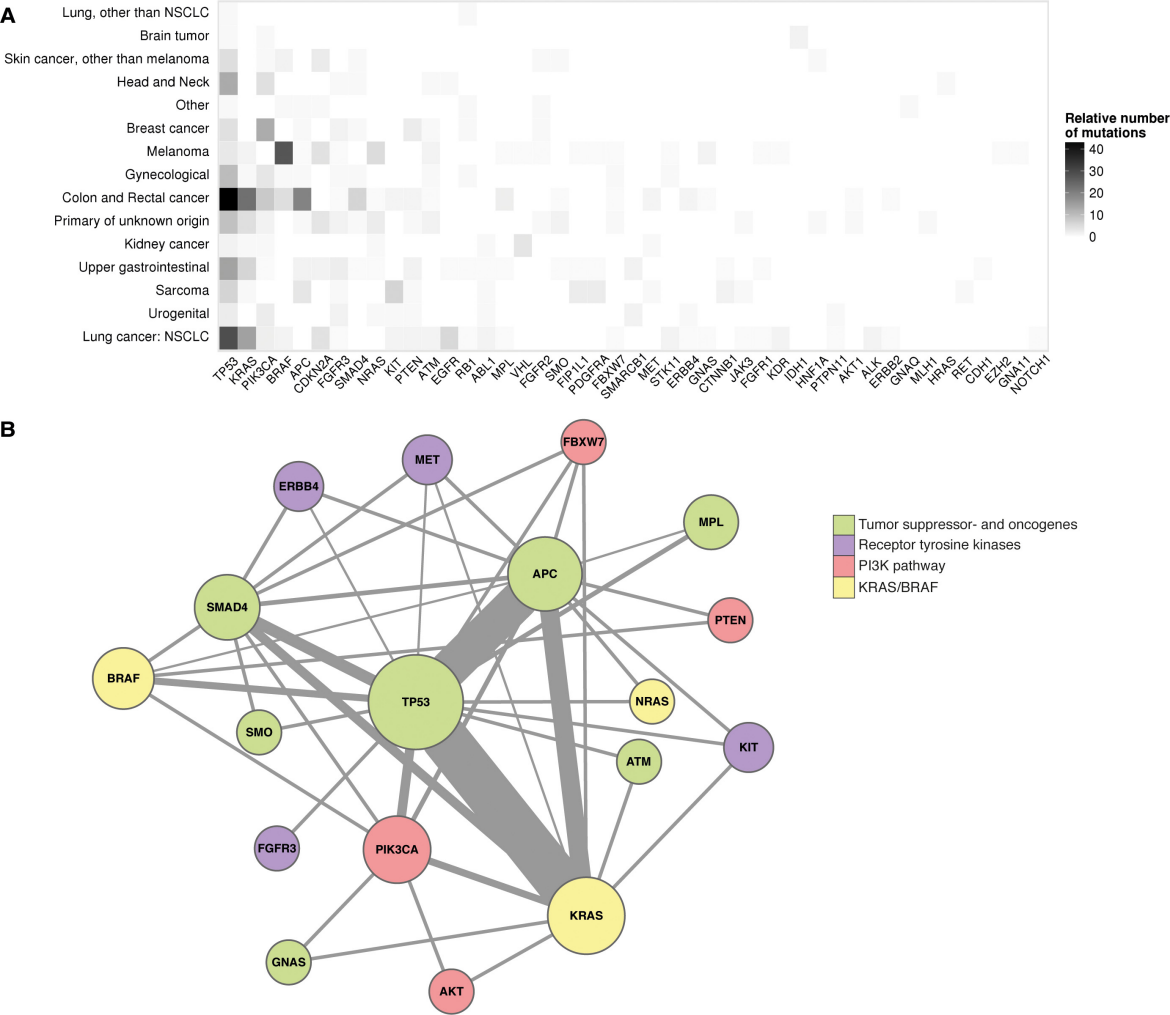
Variant distribution of the complete dataset. A) Heatmap of number of variants per tumour group. On the y-axis the different primary tumour site is depicted and on the x-axis all genes with mutational data are depicted. The relative number of mutations is defined as the number of mutations normalized per number of samples in the tumour group. B) co-occurrence of different variants in colorectal tumours. The size of the circle around a gene is indicative of the number of times a variant is identified in the gene. The lines represent co-occurrences between genes where the line thickness indicates the number of co-occurrences. The colour of the circles indicates the function of the gene: green–tumour suppressor genes and oncogenes, purple–receptor tyrosine kinases, pink–PI3K pathway, yellow–KRAS/BRAF pathway.

For all samples site of tumour origin was used to analyse the frequency of mutational distribution among the different tumour types. As expected, *TP53* was found to be the most frequently mutated gene in this unselected set of tumours (Fig 7A). The dataset contains a sample bias towards colorectal cancer, non-small cell lung carcinoma (NSCLC) and melanoma probably caused by the fact that in these tumour types mutational data already influences therapy choice, and clinicians are therefore more likely to request NGS analysis in patients with such tumours (S7 Fig).

## Discussion

Broad application of NGS for “druggable” mutation detection in a diagnostic setting relies on several aspects including the possibility to use FFPE material, fast turnaround time and stable performance over time. In this study we explored whether a targeted multi gene NGS assay can be used for diagnostic purposes in a clinical oncology setting. It comprises a 50-gene hotspot panel for the Ion Torrent platform with an average coverage depth of 1000x that was well able to derive informative data from DNA extracted from FFPE tissue. The requirements for input material are relatively minor (20 ng DNA from samples with at least 10% tumour cell percentage) generating reproducible data with a detection limit of 5% allele frequency within 5 days. There was no clear correlation between tumour cell percentage and allele frequency of the called variants above a tumour cell percentage of 10%, implying that tumour percentage is inherently difficult to interpret. As it lacks a gold standard, we feel that traditional pathology review in NGS based analysis is for determining the fact that there is >10% presence of tumour cells rather than the exact percentage of tumour cells.

It is increasingly appreciated that small genetic sub-clones within a tumour can underlie resistance,[30–32] therefore, detecting such low-abundance mutations can be of great importance. It seems furthermore feasible to implement the Ion Torrent platform for ‘liquid biopsies’[33] in which plasma cell-free circulating tumour DNA can be extracted from a blood sample to detect tumour mutations.

The process of formalin fixation and paraffin embedding induces chemical modifications, cross-linking and fragmentation of DNA.[34–37] As a result, DNA isolated from archived FFPE samples may be of poor quality, which may result in incomplete to even unreliable target amplification. However, in the present study we have shown comparable sequencing results for both FF as well as FFPE samples, when fixed according to a standardized protocol, although we found a trend in decreasing mean coverage depth with increasing amplicon length for FFPE samples only where amplicon length is stable up to 100 bp. For amplicons above 140 bp (*STK11* and *RET*) mean coverage depth clearly decreases below 1,000x. Therefore, amplicon length should be taken into account in the design of future NGS gene panels. The process of formalin fixation and paraffin embedding is known to lead to C>T or G>A base transitions, causing non-reproducible sequence alterations.[27, 28, 38] Our results support the fact that FFPE induced DNA damage appears to be minimal using this targeted hotspot approach, which has also been suggested elsewhere.[39]

Cross-validation of the Ion Torrent based NGS results using classical sequencing methods yielded a sensitivity of 98.5% which could even be improved upon manual inspection of sequence data. Data obtained were in line with results in other studies examining incidence of mutations across various tumour types. As expected, mutual exclusion of *BRAF*, *KRAS* and *NRAS* mutations was also identified in our dataset (Fig 7B). The interaction plot clearly confirmed the central role for *TP53*, *KRAS* and *APC* in colorectal tumorigenesis and depicts potential combinations of mutations that could indicate less frequent subtypes of colorectal tumours. This type of data analysis on extended datasets could help to define clinically important subtypes of tumours that we are currently unable to define using standard diagnostic tools.

Evaluation of mutations in several genes and exons is already standard in current practice. For metastatic colorectal and lung cancer patients, where treatment with anti-EGFR monoclonal antibodies is only effective in patients whose tumours are RAS wild type, sequencing 3 exons of *KRAS* and 3 exons of *NRAS* is required, and this will possibly also be the case for other tumour types shortly. The detection of copy number aberrations[40] and translocations, which are also of major clinical relevance, may be feasible in the near future using a targeted NGS approach. Personalized treatment approaches are actively exploring combinations of genetic properties (https://clinicaltrials.gov/). Thus, the need for easy to implement and flexible multi-gene tests is increasing. The easy addition of extra amplicons to these gene panels provides this assay with the flexibility needed in light of the fast discovery of new targetable mutations in cancer diagnostics.

In conclusion, NGS based diagnostics on both FF and FFPE tissue samples can be implemented in the routine clinical setting. Our study provides a guideline for standardized NGS data annotation using benchtop sequencers combined with commercially available gene panels.

## Supporting Information

S1 Fig Characteristics of tumour groups. Bar graph of the number of patients per tumour group for the method validation set in orange and the number of patients per tumour group for the test set in blue.
(TIF)

S2 Fig Average mean coverage of the NGS gene panel. Per amplicon in the NGS panel the average mean coverage for FF and FFPE samples is described. The orange line indicates the mean coverage threshold of 800x.
(TIF)

S3 Fig Changes in run statistics in time. The percentage of ISP density, low quality reads, polyclonals and usable reads is depicted for all runs (sorted by run date). Runs containing FF samples show a stable linear regression line for all run statistics. Runs containing FFPE samples show a decrease in the ISP density and usable reads percentages and an increase in the percentage of polyclonals.
(TIF)

S4 Fig Average mean coverage per length bin. All Ampliseq Cancer Hotspot Panel v2 amplicons were divided in length bins. Average mean coverage for FFPE and FF samples per length bin is depicted.
(TIF)

S5 Fig Frequency of different base substitutions for FFPE and fresh frozen samples. Formalin fixation is known to induce cross-linking of cytosines resulting in a base substitution to a thymine (C:G>T:A). Using a Chi-squared test, no significant difference in the distribution of the different base substitutions is identified in FFPE versus FF samples.
(TIF)

S6 Fig Sensitivity of Ion Torrent runs. Expected and observed frequencies of diluted TP53 variants are plotted. Observed and expected frequencies are very similar indicating that the Ion Torrent is a very sensitive method that can reliably detect variants with a frequency of 2%.
(TIF)

S7 Fig Characteristics of tumour groups. Bar graph of the relative number of variants (normalized for tumour group size) per tumour group for the validation set.
(TIF)

S8 Fig TP53 variant comparison. Variants identified in the TCGA database are compared to the variants described in this study. A) *TP53* variant distribution of the TCGA for comparison with Fig 6A that contains the TP53 variants identified in this study. B) Venn diagram showing that the 82% of the TP53 variants identified in this study are also mentioned in the TCGA database.
(TIF)

S1 Table Gene and exon information of conventional techniques. (XLSX)

S2 Table NGS sample characteristics and sequencing results. Sequencing data of all samples described in this study are visualized in this table. Furthermore, tumour type and patient number is added to allow identification of multiple tumours from the same patient.
(XLSX)

S3 Table Settings torrent variant caller. Parameter settings used by the Torrent Variant Caller for calling somatic variants. For FF samples low stringency settings were used and for FFPE samples high stringency settings were used.
(XLSX)

S4 Table Explanation of different results between conventional techniques and NGS. For *TP53* the Ampliseq panel provided less information since the amplicon pool does not cover the same sequence compared to conventional techniques. NGS coverage of other genes was improved compared to the conventional technique providing extra information; 2 *KRAS* exon 4 and 1 *TP53* exon 10 mutations were identified in regions that were not tested with the conventional techniques. In 2 samples a *BRAF* exon 15 (p.V600K) was identified where the Cobas technique only provided information on the presence of a mutation, but did not specify which mutation.
(XLSX)

## References

[1] Wheeler DA, Srinivasan M, Egholm M, Shen Y, Chen L, McGuire A, et al The complete genome of an individual by massively parallel DNA sequencing. Nature. 2008;452(7189):872–6. Epub 2008/04/19. doi: nature06884 [pii] 10.1038/nature06884 . 18421352

[2] Martinez DA, Nelson MA. The next generation becomes the now generation. PLoS Genet. 2010;6(4):e1000906 Epub 2010/04/14. 10.1371/journal.pgen.1000906 20386747 PMC2851573

[3] Hinrichs JW, van Blokland WT, Moons MJ, Radersma RD, Radersma-van Loon JH, de Voijs CM, et al Comparison of next-generation sequencing and mutation-specific platforms in clinical practice. Am J Clin Pathol. 2015;143(4):573–8. Epub 2015/03/18. doi: 143/4/573 [pii] 10.1309/AJCP40XETVYAMJPY . 25780010

[4] Diaz LA Jr., Williams RT, Wu J, Kinde I, Hecht JR, Berlin J, et al The molecular evolution of acquired resistance to targeted EGFR blockade in colorectal cancers. Nature. 2012;486(7404):537–40. Epub 2012/06/23. doi: nature11219 [pii] 10.1038/nature11219 22722843 PMC3436069

[5] Loman NJ, Misra RV, Dallman TJ, Constantinidou C, Gharbia SE, Wain J, et al Performance comparison of benchtop high-throughput sequencing platforms. Nat Biotechnol. 2012;30(5):434–9. Epub 2012/04/24. doi: nbt.2198 [pii] 10.1038/nbt.2198 . 22522955

[6] Pant S, Weiner R, Marton MJ. Navigating the rapids: the development of regulated next-generation sequencing-based clinical trial assays and companion diagnostics. Front Oncol. 2014;4:78 Epub 2014/05/27. 10.3389/fonc.2014.00078 24860780 PMC4029014

[7] Cottrell CE, Al-Kateb H, Bredemeyer AJ, Duncavage EJ, Spencer DH, Abel HJ, et al Validation of a next-generation sequencing assay for clinical molecular oncology. J Mol Diagn. 2014;16(1):89–105. Epub 2013/11/12. doi: S1525-1578(13)00219-5 [pii] 10.1016/j.jmoldx.2013.10.002 . 24211365 PMC5762937

[8] Frampton GM, Fichtenholtz A, Otto GA, Wang K, Downing SR, He J, et al Development and validation of a clinical cancer genomic profiling test based on massively parallel DNA sequencing. Nat Biotechnol. 2013;31(11):1023–31. Epub 2013/10/22. doi: nbt.2696 [pii] 10.1038/nbt.2696 . 24142049 PMC5710001

[9] Quail MA, Smith M, Coupland P, Otto TD, Harris SR, Connor TR, et al A tale of three next generation sequencing platforms: comparison of Ion Torrent, Pacific Biosciences and Illumina MiSeq sequencers. BMC genomics. 2012;13:341 Epub 2012/07/26. 10.1186/1471-2164-13-341 22827831 PMC3431227

[10] Gleeson FC, Kipp BR, Kerr SE, Voss JS, Graham RP, Campion MB, et al Kinase genotype analysis of gastric gastrointestinal stromal tumor cytology samples using targeted next-generation sequencing. Clinical gastroenterology and hepatology: the official clinical practice journal of the American Gastroenterological Association. 2015;13(1):202–6. Epub 2014/07/06. 10.1016/j.cgh.2014.06.024 . 24997326

[11] Ihle MA, Fassunke J, Konig K, Grunewald I, Schlaak M, Kreuzberg N, et al Comparison of high resolution melting analysis, pyrosequencing, next generation sequencing and immunohistochemistry to conventional Sanger sequencing for the detection of p.V600E and non-p.V600E BRAF mutations. BMC Cancer. 2014;14:13. Epub 2014/01/15. doi: 1471-2407-14-13 [pii] 10.1186/1471-2407-14-13 24410877 PMC3893431

[12] Sie D, Snijders PJ, Meijer GA, Doeleman MW, van Moorsel MI, van Essen HF, et al Performance of amplicon-based next generation DNA sequencing for diagnostic gene mutation profiling in oncopathology. Cell Oncol (Dordr). 2014;37(5):353–61. Epub 2014/09/12. 10.1007/s13402-014-0196-2 . 25209392 PMC13004472

[13] Hagemann IS, Devarakonda S, Lockwood CM, Spencer DH, Guebert K, Bredemeyer AJ, et al Clinical next-generation sequencing in patients with non-small cell lung cancer. Cancer. 2015;121(4):631–9. Epub 2014/10/28. 10.1002/cncr.29089 . 25345567

[14] Johnson DB, Dahlman KH, Knol J, Gilbert J, Puzanov I, Means-Powell J, et al Enabling a genetically informed approach to cancer medicine: a retrospective evaluation of the impact of comprehensive tumor profiling using a targeted next-generation sequencing panel. The oncologist. 2014;19(6):616–22. Epub 2014/05/07. 10.1634/theoncologist.2014-0011 24797823 PMC4041676

[15] Belardinilli F, Capalbo C, Buffone A, Petroni M, Colicchia V, Ferraro S, et al Validation of the Ion Torrent PGM sequencing for the prospective routine molecular diagnostic of colorectal cancer. Clinical biochemistry. 2015;48(13–14):908–10. Epub 2015/04/15. 10.1016/j.clinbiochem.2015.04.003 . 25872148

[16] Malapelle U, Vigliar E, Sgariglia R, Bellevicine C, Colarossi L, Vitale D, et al Ion Torrent next-generation sequencing for routine identification of clinically relevant mutations in colorectal cancer patients. J Clin Pathol. 2015;68(1):64–8. Epub 2014/11/08. 10.1136/jclinpath-2014-202691 . 25378536

[17] Lin MT, Mosier SL, Thiess M, Beierl KF, Debeljak M, Tseng LH, et al Clinical validation of KRAS, BRAF, and EGFR mutation detection using next-generation sequencing. Am J Clin Pathol. 2014;141(6):856–66. Epub 2014/05/20. doi: AJCPMWGWGO34EGOD [pii] 10.1309/AJCPMWGWGO34EGOD 24838331 PMC4332779

[18] Mafficini A, Amato E, Fassan M, Simbolo M, Antonello D, Vicentini C, et al Reporting tumor molecular heterogeneity in histopathological diagnosis. PLoS One. 2014;9(8):e104979 Epub 2014/08/16. 10.1371/journal.pone.0104979 PONE-D-14-16410 [pii]. 25127237 PMC4134249

[19] McCourt CM, McArt DG, Mills K, Catherwood MA, Maxwell P, Waugh DJ, et al Validation of next generation sequencing technologies in comparison to current diagnostic gold standards for BRAF, EGFR and KRAS mutational analysis. PLoS One. 2013;8(7):e69604 Epub 2013/08/08. 10.1371/journal.pone.0069604 PONE-D-13-05730 [pii]. 23922754 PMC3724913

[20] Portier BP, Kanagal-Shamanna R, Luthra R, Singh R, Routbort MJ, Handal B, et al Quantitative assessment of mutant allele burden in solid tumors by semiconductor-based next-generation sequencing. Am J Clin Pathol. 2014;141(4):559–72. Epub 2014/03/13. doi: 141/4/559 [pii] 10.1309/AJCP1JUGQMW7ZNTL . 24619758

[21] Li H, Durbin R. Fast and accurate short read alignment with Burrows-Wheeler transform. Bioinformatics. 2009;25(14):1754–60. Epub 2009/05/20. 10.1093/bioinformatics/btp324 19451168 PMC2705234

[22] Li H, Durbin R. Fast and accurate long-read alignment with Burrows-Wheeler transform. Bioinformatics. 2010;26(5):589–95. Epub 2010/01/19. 10.1093/bioinformatics/btp698 20080505 PMC2828108

[23] Ning Z, Cox AJ, Mullikin JC. SSAHA: a fast search method for large DNA databases. Genome research. 2001;11(10):1725–9. Epub 2001/10/10. 10.1101/gr.194201 11591649 PMC311141

[24] Li H. Exploring single-sample SNP and INDEL calling with whole-genome de novo assembly. Bioinformatics. 2012;28(14):1838–44. Epub 2012/05/10. 10.1093/bioinformatics/bts280 22569178 PMC3389770

[25] McLaren W, Pritchard B, Rios D, Chen Y, Flicek P, Cunningham F. Deriving the consequences of genomic variants with the Ensembl API and SNP Effect Predictor. Bioinformatics. 2010;26(16):2069–70. Epub 2010/06/22. doi: btq330 [pii] 10.1093/bioinformatics/btq330 20562413 PMC2916720

[26] Robinson JT, Thorvaldsdottir H, Winckler W, Guttman M, Lander ES, Getz G, et al Integrative genomics viewer. Nat Biotechnol. 2011;29(1):24–6. Epub 2011/01/12. doi: nbt.1754 [pii] 10.1038/nbt.1754 21221095 PMC3346182

[27] Srinivasan M, Sedmak D, Jewell S. Effect of fixatives and tissue processing on the content and integrity of nucleic acids. Am J Pathol. 2002;161(6):1961–71. Epub 2002/12/06. doi: S0002-9440(10)64472-0 [pii] 10.1016/S0002-9440(10)64472-0 12466110 PMC1850907

[28] Williams C, Ponten F, Moberg C, Soderkvist P, Uhlen M, Ponten J, et al A high frequency of sequence alterations is due to formalin fixation of archival specimens. Am J Pathol. 1999;155(5):1467–71. Epub 1999/11/07. doi: S0002-9440(10)65461-2 [pii] 10.1016/S0002-9440(10)65461-2 10550302 PMC1866966

[29] Smits AJ, Kummer JA, de Bruin PC, Bol M, van den Tweel JG, Seldenrijk KA, et al The estimation of tumor cell percentage for molecular testing by pathologists is not accurate. Mod Pathol. 2014;27(2):168–74. Epub 2013/07/28. doi: modpathol2013134 [pii] 10.1038/modpathol.2013.134 . 23887293

[30] Inukai M, Toyooka S, Ito S, Asano H, Ichihara S, Soh J, et al Presence of epidermal growth factor receptor gene T790M mutation as a minor clone in non-small cell lung cancer. Cancer Res. 2006;66(16):7854–8. Epub 2006/08/17. doi: 66/16/7854 [pii] 10.1158/0008-5472.CAN-06-1951 . 16912157

[31] Maheswaran S, Sequist LV, Nagrath S, Ulkus L, Brannigan B, Collura CV, et al Detection of mutations in EGFR in circulating lung-cancer cells. N Engl J Med. 2008;359(4):366–77. Epub 2008/07/04. doi: NEJMoa0800668 [pii] 10.1056/NEJMoa0800668 18596266 PMC3551471

[32] Taniguchi K, Okami J, Kodama K, Higashiyama M, Kato K. Intratumor heterogeneity of epidermal growth factor receptor mutations in lung cancer and its correlation to the response to gefitinib. Cancer Sci. 2008;99(5):929–35. Epub 2008/03/08. doi: CAS782 [pii] 10.1111/j.1349-7006.2008.00782.x . 18325048 PMC11158886

[33] Rothe F, Laes JF, Lambrechts D, Smeets D, Vincent D, Maetens M, et al Plasma circulating tumor DNA as an alternative to metastatic biopsies for mutational analysis in breast cancer. Ann Oncol. 2014;25(10):1959–65. Epub 2014/09/04. doi: mdu288 [pii] 10.1093/annonc/mdu288 . 25185240

[34] Blow N. Tissue preparation: Tissue issues. Nature. 2007;448(7156):959–63. Epub 2007/08/24. doi: 448959a [pii] 10.1038/448959a . 17713539

[35] Dietrich D, Uhl B, Sailer V, Holmes EE, Jung M, Meller S, et al Improved PCR performance using template DNA from formalin-fixed and paraffin-embedded tissues by overcoming PCR inhibition. PLoS One. 2013;8(10):e77771 Epub 2013/10/25. 10.1371/journal.pone.0077771 PONE-D-13-18657 [pii]. 24155973 PMC3796491

[36] Hedegaard J, Thorsen K, Lund MK, Hein AM, Hamilton-Dutoit SJ, Vang S, et al Next-generation sequencing of RNA and DNA isolated from paired fresh-frozen and formalin-fixed paraffin-embedded samples of human cancer and normal tissue. PLoS One. 2014;9(5):e98187 Epub 2014/06/01. 10.1371/journal.pone.0098187 PONE-D-14-11652 [pii]. 24878701 PMC4039489

[37] Latham GJ. Next-generation sequencing of formalin-fixed, paraffin-embedded tumor biopsies: navigating the perils of old and new technology to advance cancer diagnosis. Expert Rev Mol Diagn. 2013;13(8):769–72. Epub 2013/10/15. 10.1586/14737159.2013.845090 . 24117229

[38] Xuan J, Yu Y, Qing T, Guo L, Shi L. Next-generation sequencing in the clinic: promises and challenges. Cancer Lett. 2013;340(2):284–95. Epub 2012/11/24. doi: S0304-3835(12)00672-6 [pii] 10.1016/j.canlet.2012.11.025 . 23174106 PMC5739311

[39] Spencer DH, Sehn JK, Abel HJ, Watson MA, Pfeifer JD, Duncavage EJ. Comparison of clinical targeted next-generation sequence data from formalin-fixed and fresh-frozen tissue specimens. J Mol Diagn. 2013;15(5):623–33. Epub 2013/07/03. doi: S1525-1578(13)00091-3 [pii] 10.1016/j.jmoldx.2013.05.004 . 23810758 PMC4912568

[40] Hoogstraat M, Hinrichs JW, Besselink NJ, Radersma-van Loon JH, de Voijs CM, Peeters T, et al Simultaneous detection of clinically relevant mutations and amplifications for routine cancer pathology. J Mol Diagn. 2015;17(1):10–8. Epub 2014/12/03. doi: S1525-1578(14)00177-9 [pii] 10.1016/j.jmoldx.2014.09.004 . 25445215

