# Supplementary figures and images for: Targeted Next Generation Sequencing as a Reliable Diagnostic Assay for the Detection of Somatic Mutations in Tumours Using Minimal DNA Amounts from Formalin Fixed Paraffin Embedded Material
Source: PLoS One. 2016 Feb 26;11(2):e0149405. doi: 10.1371/journal.pone.0149405 (PMC4769293; doi:10.1371/journal.pone.0149405)

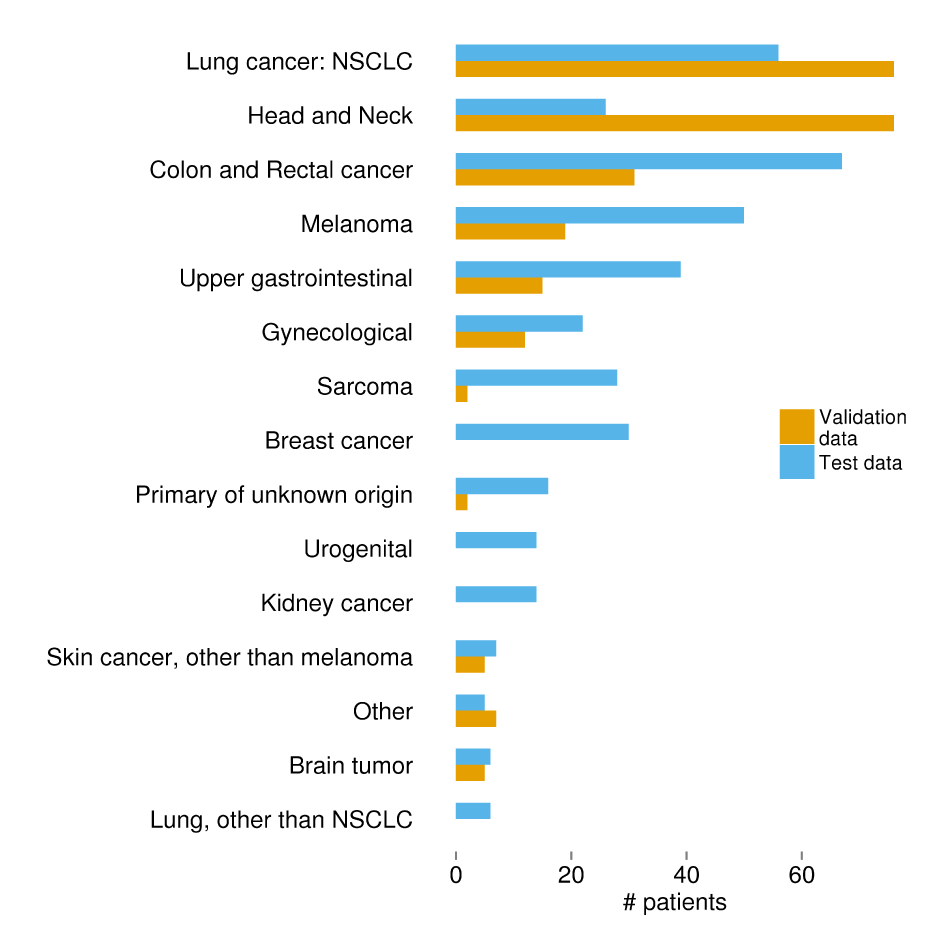

Supplement: S1 Fig — Bar graph of the number of patients per tumour group for the method validation set in orange and the number of patients per tumour group for the test set in blue. (TIF) [file pone.0149405.s001.tif]

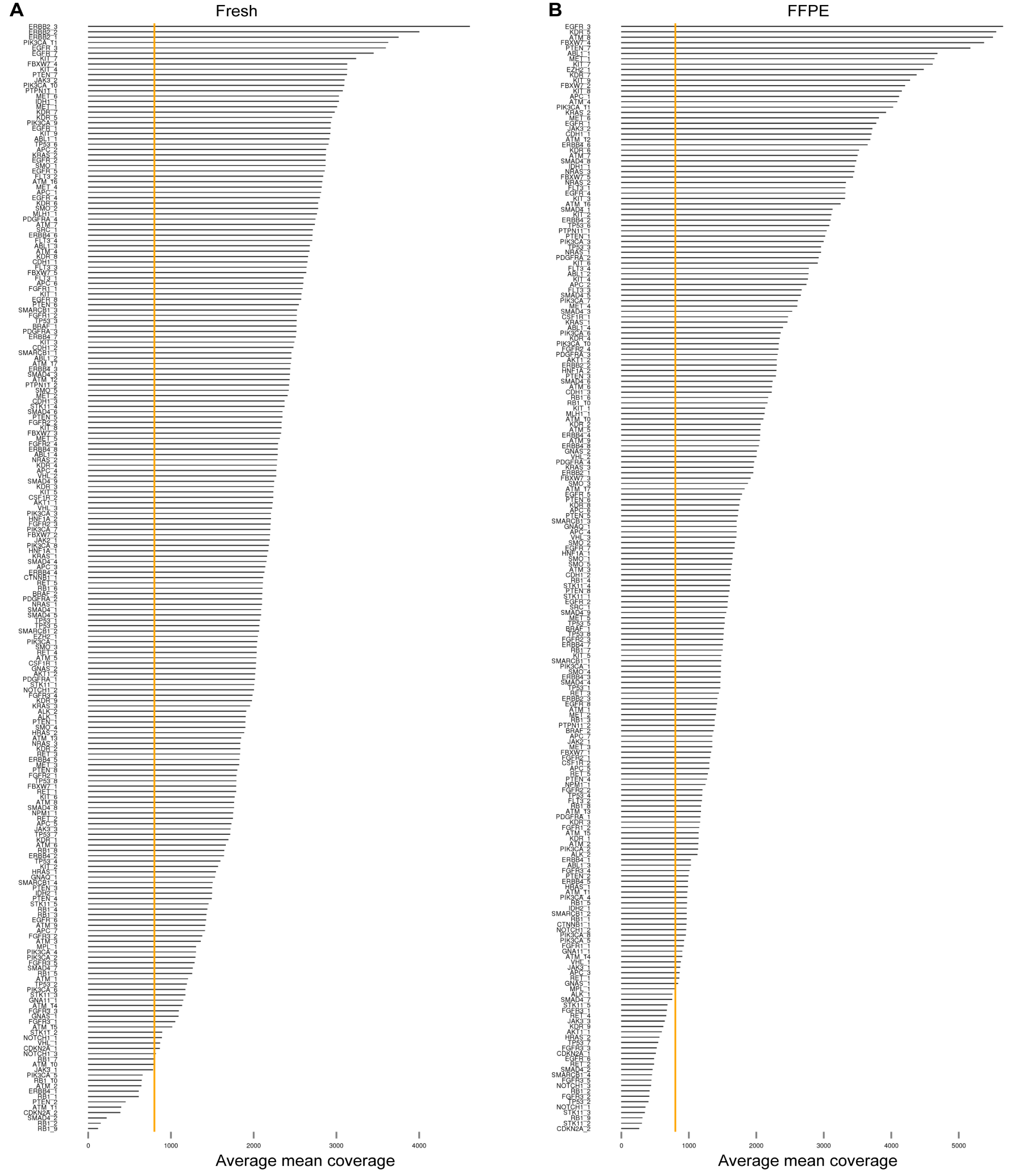

Supplement: S2 Fig — Per amplicon in the NGS panel the average mean coverage for FF and FFPE samples is described. The orange line indicates the mean coverage threshold of 800x. (TIF) [file pone.0149405.s002.tif]

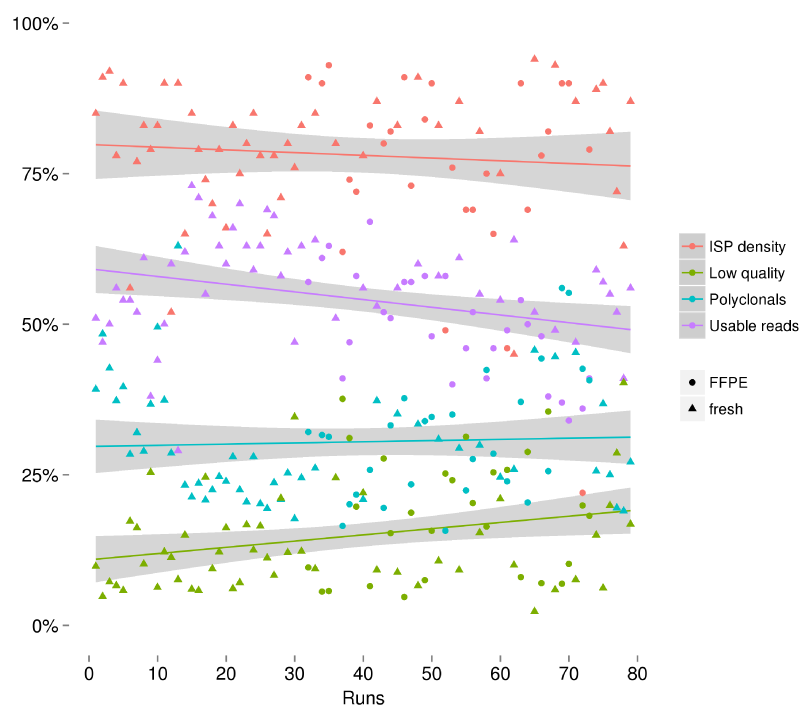

Supplement: S3 Fig — The percentage of ISP density, low quality reads, polyclonals and usable reads is depicted for all runs (sorted by run date). Runs containing FF samples show a stable linear regression line for all run statistics. Runs containing FFPE samples show a decrease in the ISP density and usable reads percentages and an increase in the percentage of polyclonals. (TIF) [file pone.0149405.s003.tif]

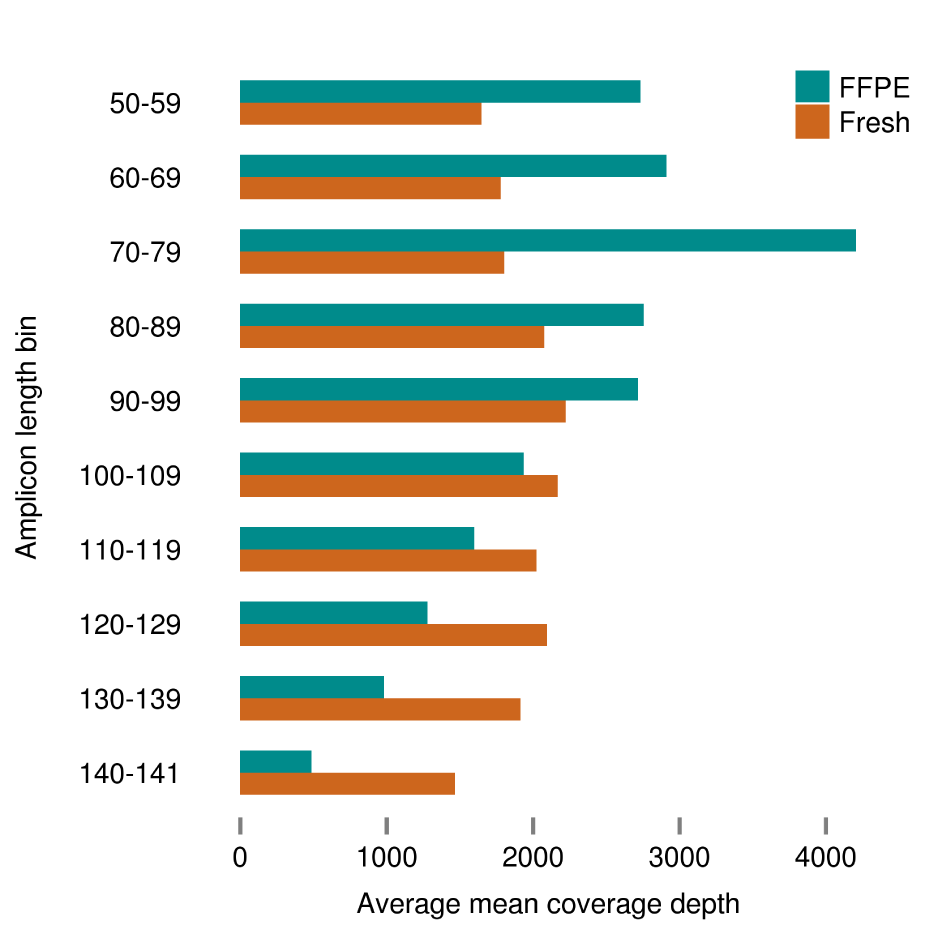

Supplement: S4 Fig — All Ampliseq Cancer Hotspot Panel v2 amplicons were divided in length bins. Average mean coverage for FFPE and FF samples per length bin is depicted. (TIF) [file pone.0149405.s004.tif]

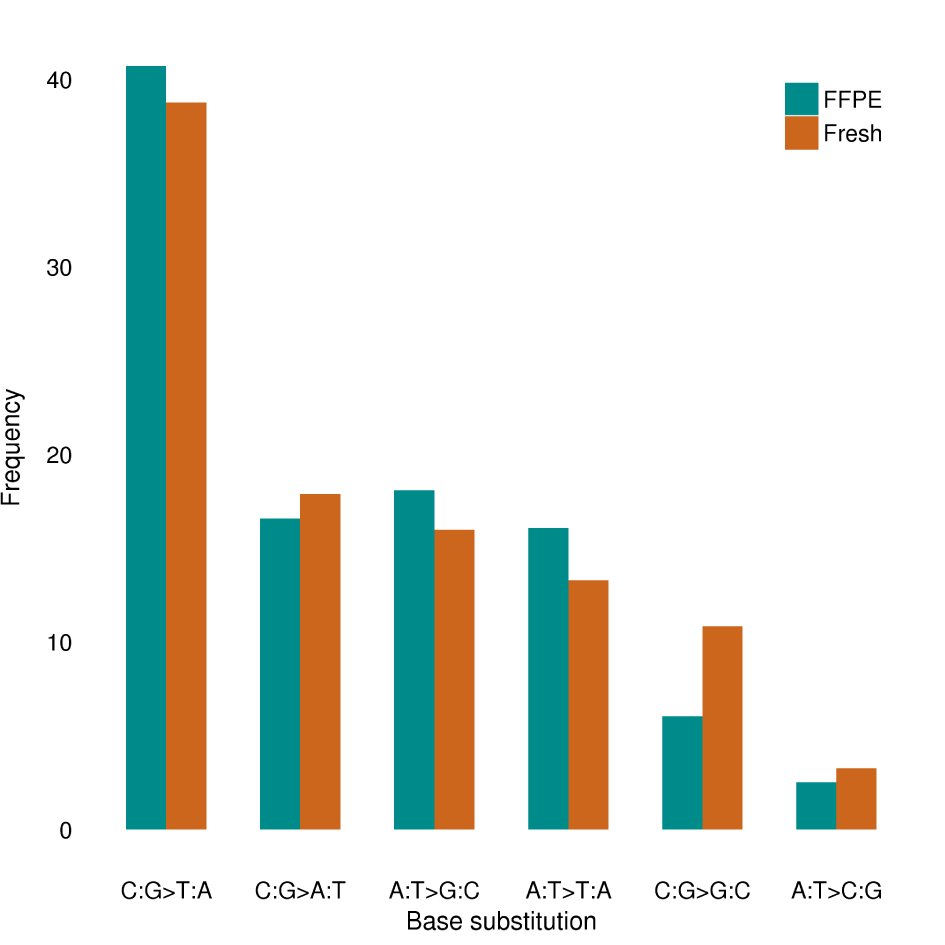

Supplement: S5 Fig — Formalin fixation is known to induce cross-linking of cytosines resulting in a base substitution to a thymine (C:G>T:A). Using a Chi-squared test, no significant difference in the distribution of the different base substitutions is identified in FFPE versus FF samples. (TIF) [file pone.0149405.s005.tif]

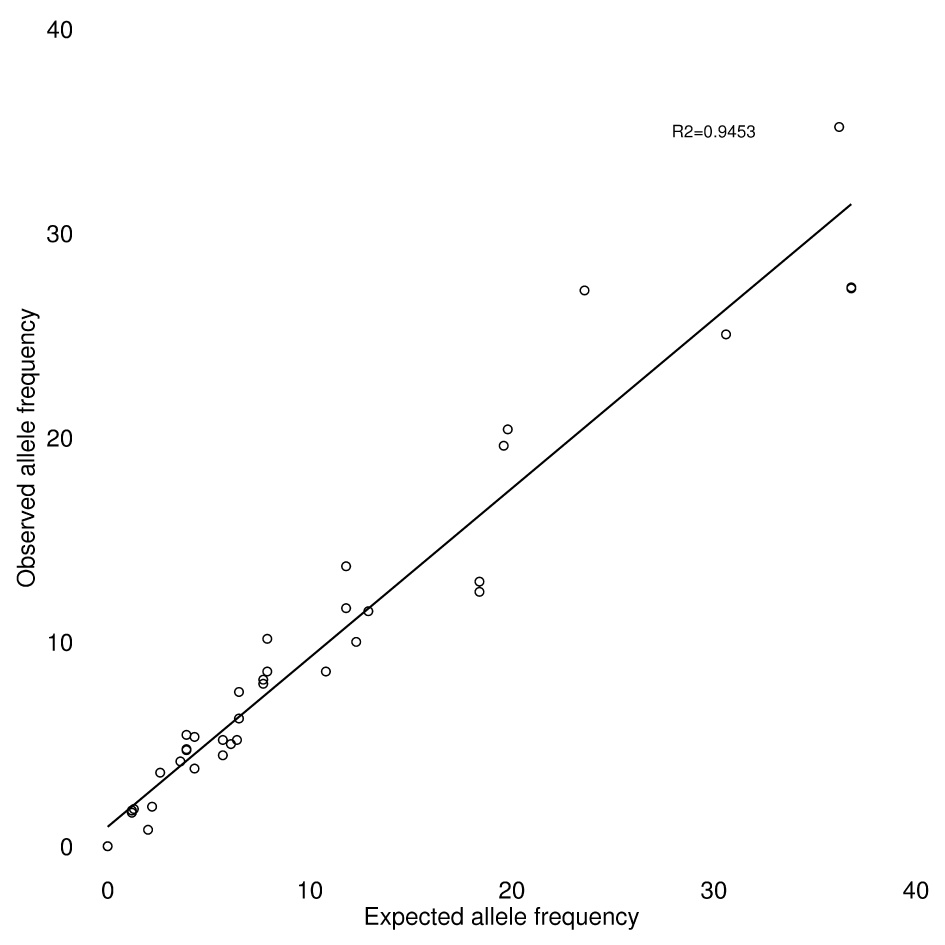

Supplement: S6 Fig — Expected and observed frequencies of diluted TP53 variants are plotted. Observed and expected frequencies are very similar indicating that the Ion Torrent is a very sensitive method that can reliably detect variants with a frequency of 2%. (TIF) [file pone.0149405.s006.tif]

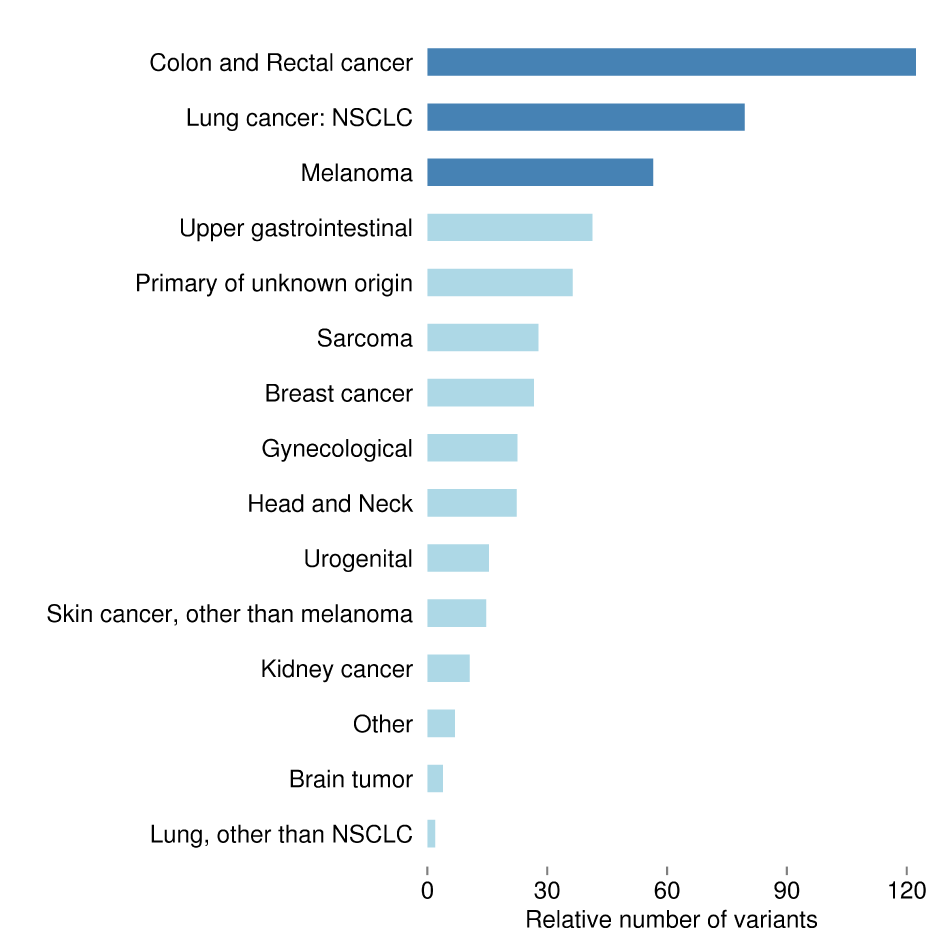

Supplement: S7 Fig — Bar graph of the relative number of variants (normalized for tumour group size) per tumour group for the validation set. (TIF) [file pone.0149405.s007.tif]

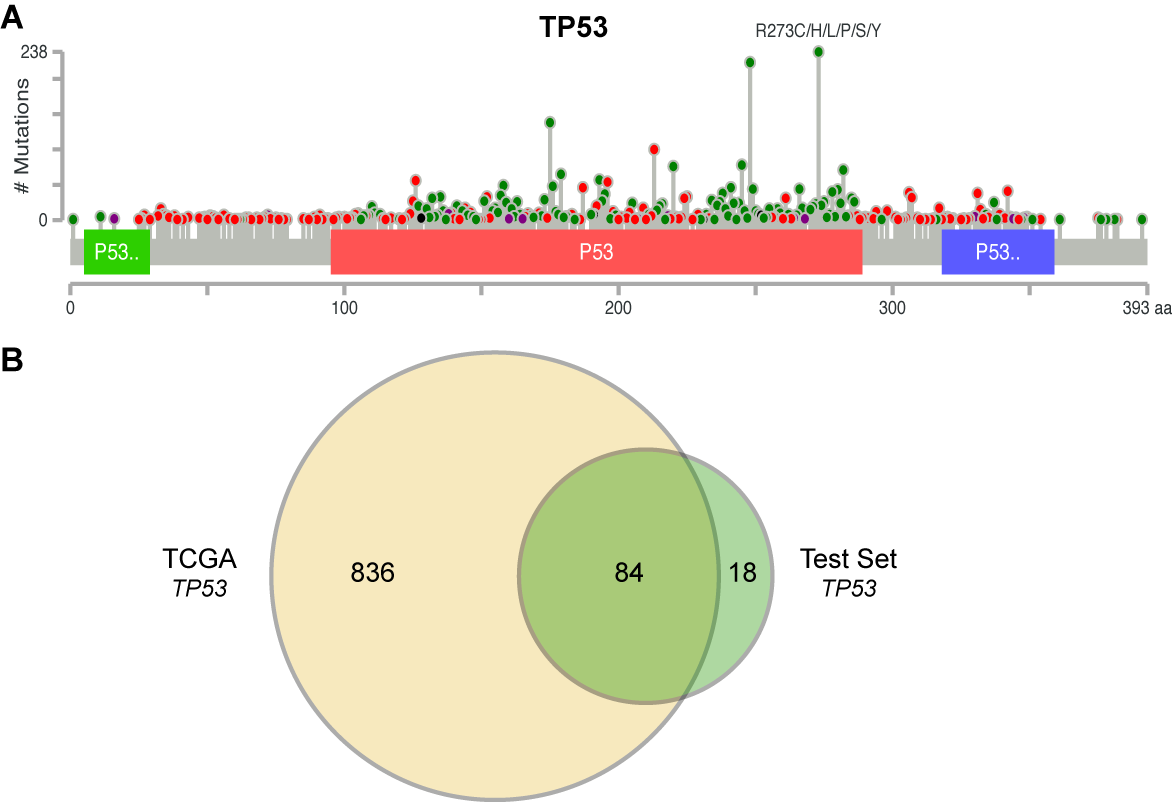

Supplement: S8 Fig — Variants identified in the TCGA database are compared to the variants described in this study. A) TP53 variant distribution of the TCGA for comparison with Fig 6A that contains the TP53 variants identified in this study. B) Venn diagram showing that the 82% of the TP53 variants identified in this study are also mentioned in the TCGA database. (TIF) [file pone.0149405.s008.tif]
